# Anionic N_18_ Macrocycles and a Polynitrogen Double Helix in Novel Yttrium Polynitrides YN_6_ and Y_2_N_11_ at 100 GPa

**DOI:** 10.1002/anie.202207469

**Published:** 2022-07-13

**Authors:** Andrey Aslandukov, Florian Trybel, Alena Aslandukova, Dominique Laniel, Timofey Fedotenko, Saiana Khandarkhaeva, Georgios Aprilis, Carlotta Giacobbe, Eleanor Lawrence Bright, Igor A. Abrikosov, Leonid Dubrovinsky, Natalia Dubrovinskaia

**Affiliations:** ^1^ Material Physics and Technology at Extreme Conditions Laboratory of Crystallography University of Bayreuth Universitätstrasse 30 95440 Bayreuth Germany; ^2^ Bayerisches Geoinstitut University of Bayreuth Universitätstrasse 30 95440 Bayreuth Germany; ^3^ Department of Physics Chemistry and Biology (IFM) Linköping University 58183 Linköping Sweden; ^4^ Centre for Science at Extreme Conditions and School of Physics and Astronomy University of Edinburgh Edinburgh EH9 3FD UK; ^5^ Photon Science, Deutsches Elektronen-Synchrotron Notkestrasse 85 22607 Hamburg Germany; ^6^ European Synchrotron Radiation Facility BP 220 38043 Grenoble Cedex France

**Keywords:** High-Pressure Chemistry, Inorganic Double Helix, Macrocycles, Polynitrides

## Abstract

Two novel yttrium nitrides, YN_6_ and Y_2_N_11_, were synthesized by direct reaction between yttrium and nitrogen at 100 GPa and 3000 K in a laser‐heated diamond anvil cell. High‐pressure synchrotron single‐crystal X‐ray diffraction revealed that the crystal structures of YN_6_ and Y_2_N_11_ feature a unique organization of nitrogen atoms—a previously unknown anionic N_18_ macrocycle and a polynitrogen double helix, respectively. Density functional theory calculations, confirming the dynamical stability of the YN_6_ and Y_2_N_11_ compounds, show an anion‐driven metallicity, explaining the unusual bond orders in the polynitrogen units. As the charge state of the polynitrogen double helix in Y_2_N_11_ is different from that previously found in Hf_2_N_11_ and because N_18_ macrocycles have never been predicted or observed, their discovery significantly extends the chemistry of polynitrides.

## Graphical abstract

The reaction between yttrium and molecular nitrogen at 100 GPa under laser heating at 3000 K yields two novel compounds, YN_6_ and Y_2_N_11_. Their crystal structures feature a unique organization of nitrogen atoms: an anionic N_18_ macrocycle and a polynitrogen double helix, respectively.

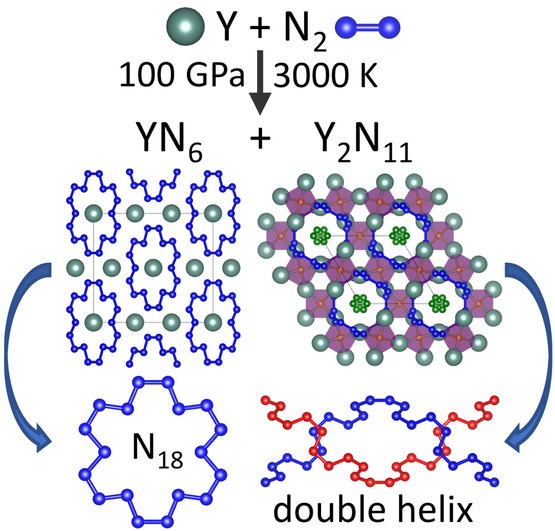

The chemistry of nitrogen has long been thought to be very limited due to triple‐bonded molecular nitrogen's extreme stability. As a result, in inorganic solid‐state compounds at ambient pressure, nitrogen is typically present in the form of a nitride anion N^3−^ and does not form catenated polyanions (with the exception of azides). However, over the past 20 years, it has been shown that at high pressure nitrogen's chemistry significantly changes. For example, charged nitrogen N_2_^*x*−^ dimers,[^1^, ^2^, ^3^, ^4^, ^5^, ^6^, ^7^, ^8^, ^9^, ^10^, ^11^, ^12^, ^13^, ^14^, ^15^, ^16^] tetranitrogen N_4_^4−^ units,^[17]^ pentazolate N_5_^−^ rings,[^18^, ^19^, ^20^] hexazine N_6_ rings,[^21^, ^22^, ^23^] and different polynitrogen chains[^17^, ^24^, ^25^, ^26^, ^27^, ^28^, ^29^] have been synthesized, and an even greater variety of nitrogen species is expected to form under high‐pressure conditions according to theoretical calculations.[^30^, ^31^, ^32^, ^33^, ^34^, ^35^, ^36^, ^37^] Such a diversity of nitrogen species can suggest that the scale of nitrogen chemistry under high pressure may be close to the scale of the rich carbon chemistry at ambient pressure. In addition to the discoveries of unique nitrogen entities that push the boundaries of fundamental nitrogen chemistry, nitrides synthesized under high pressure often possess key properties for functional applications, e.g. ReN_2_, which is recoverable to ambient conditions,^[4]^ has an extremely high hardness, a single layer of BeN_4_ is a 2D material with unique electronic properties,^[29]^ and a variety of nitrides with high nitrogen content are promising for applications as high energy density materials.^[38]^ Recently, YN_5_, YN_8,_ and YN_10_ with polynitrogen chains, fused N_18_ rings and isolated N_5_ rings, respectively, were predicted to be stable near 100 GPa and are all promising prospects as high energy density materials.^[37]^

Whereas a significant number of studies on binary metal‐nitrogen compounds of alkali, alkaline earth, and transition metal elements under high pressure have been conducted,[^1^, ^2^, ^3^, ^4^, ^5^, ^6^, ^7^, ^9^, ^10^, ^11^, ^12^, ^13^, ^14^, ^15^, ^16^, ^17^, ^18^, ^19^, ^20^, ^21^, ^22^, ^23^, ^24^, ^25^, ^26^, ^27^, ^28^, ^29^] the high‐pressure chemistry and physical properties of rare earth metal nitrides are almost unknown. Until recently, in the yttrium‐nitrogen system, only one binary Y−N compound was known: cubic yttrium nitride YN with the rock salt structure.^[39]^ In 2021 our group demonstrated that even at moderate compression (≈50 GPa) yttrium and nitrogen form a novel compound, Y_5_N_14_, with a new structural type.^[8]^ In the Y_5_N_14_ structure, all nitrogen atoms form [N_2_]^*x*−^ dimers but, strikingly, there are three crystallographically distinct nitrogen dimers with different N−N bond lengths and charge states *x*, indicating the complexity of chemical processes leading to the formation of dense rare earth metal nitrides under high pressure.

In this study, we present the synthesis and characterization of two novel never predicted yttrium nitrides YN_6_ and Y_2_N_11_ at 100 GPa, demonstrating a unique organization of nitrogen atoms in their structures, forming anionic N_18_ macrocycles and a polynitrogen double helix.

A diamond anvil cell, containing a sample composed of two pieces of yttrium embedded into molecular nitrogen, was compressed to 100(1) GPa and laser‐heated to 3000(200) K (see Figure 1a and Supporting Information for details). The precise 2D X‐ray diffraction map, collected with a step of 0.5 μm at ID11 ESRF beamline from the bigger piece of yttrium after heating, revealed the crystallization of novel phases and allowed to pinpoint the location of crystallites most appropriate for single‐crystal X‐ray diffraction measurements (Figure 1b). High‐quality synchrotron single‐crystal X‐ray diffraction (SCXRD) data were then collected from the sample (Figure 1c). The subsequent crystal structure solution and refinement revealed the formation of two novel yttrium nitrides with chemical formulas of YN_6_ and Y_2_N_11_. The refinement against SCXRD data resulted in very good reliability factors (R‐factors, see Tables S1, S2). For cross‐validation of the structural models, we performed density functional theory (DFT) based calculations (see Supporting Information for details). We carried out variable cell structural relaxations for both compounds and found that the relaxed structural parameters closely reproduce the corresponding experimental values (Table S3). The distribution of the YN_6_ and Y_2_N_11_ phases shown in the 2D X‐ray diffraction map (Figure 1b) demonstrates that the heated area consists of many tiny crystallites and there is no obvious chemical gradient in the distribution. The formation of a mixture of phases with different chemical compositions and structures is a very common phenomenon at high‐pressure synthesis in a laser‐heated diamond anvil cell mainly attributed to the temperature gradient during laser heating.

**Figure 1:**
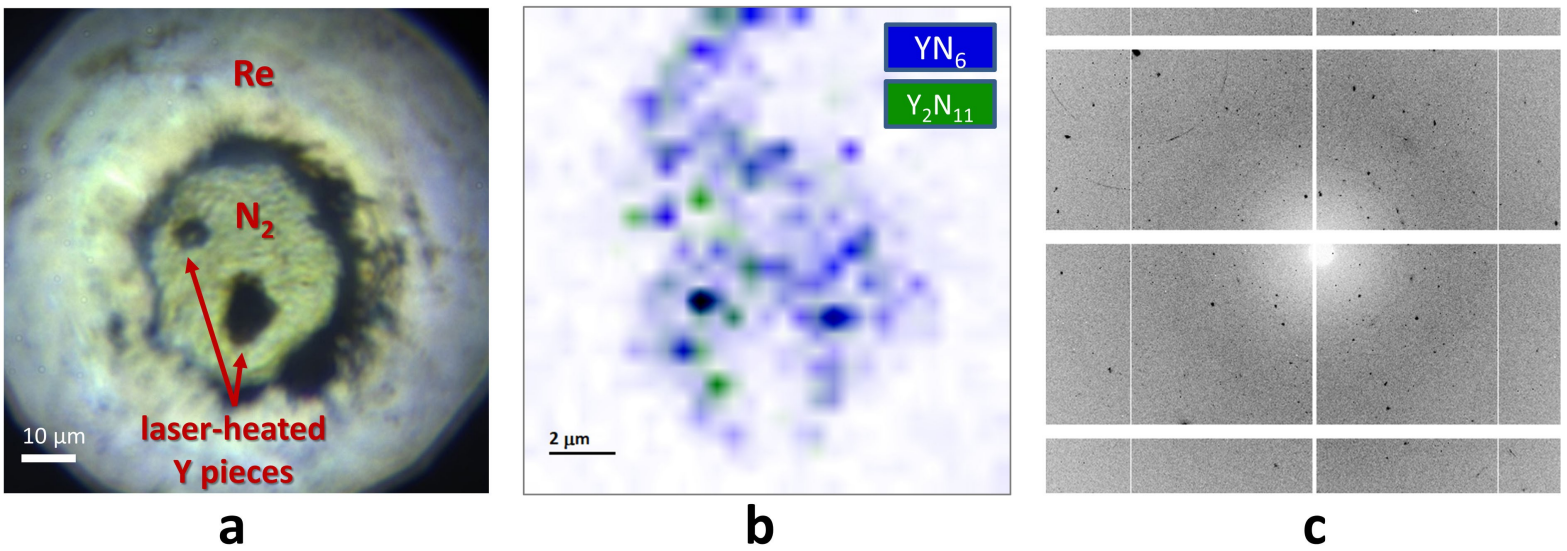
Experimental details. a) Microphotograph of the sample chamber. b) 2D X‐ray diffraction map showing the distribution of the two yttrium nitrides phases within the heated sample. The color intensity is proportional to the intensity of the following reflections: the (2 0 0), (0 2 0), and (1 1 ‐2) of YN_6_ for the blue regions; the (1 0 1), (2 ‐1 0), and (2 ‐1 4) of Y_2_N_11_ for the green regions. c) Example of an X‐ray diffraction pattern collected from the laser‐heated sample at 100 GPa.

The structure of YN_6_ (Figure 2) has the monoclinic space group *C*2/*m* (#12) with two Y and five N atoms on crystallographically distinct positions (see Table S1 and the CIF for the full crystallographic data^[40]^). Nitrogen atoms form isolated, almost planar N_18_ macrocycles aligned in the (4 0 ‐1) planes (Figure 2b). The Y1 atoms are located in the centers of the N_18_ macrocycles, while the Y2 atoms occupy the space between the stacking planes (Figure 2a). Thus, Y1 atoms are twelve‐fold coordinated (coordination number CN=12) by six nearest nitrogen atoms of the N_18_ ring itself and by three nitrogen atoms of the previous and next rings in the stack (Figure 2c). The equatorial coordination of Y1 is very similar to the coordination of the rare‐earth metals in complexes with hexaaza‐18‐membered macrocyclic ligands.[^41^, ^42^, ^43^] Y2 atoms are thirteen‐fold coordinated (CN=13) by N atoms of the six surrounding rings (Figure 2d). Both Y1 and Y2 atoms are located in the *ac* plane forming a 2D tiling which can be described by two types of parallelograms (Figure S1). The nets of Y atoms are alternating in the *b*‐direction.

**Figure 2:**
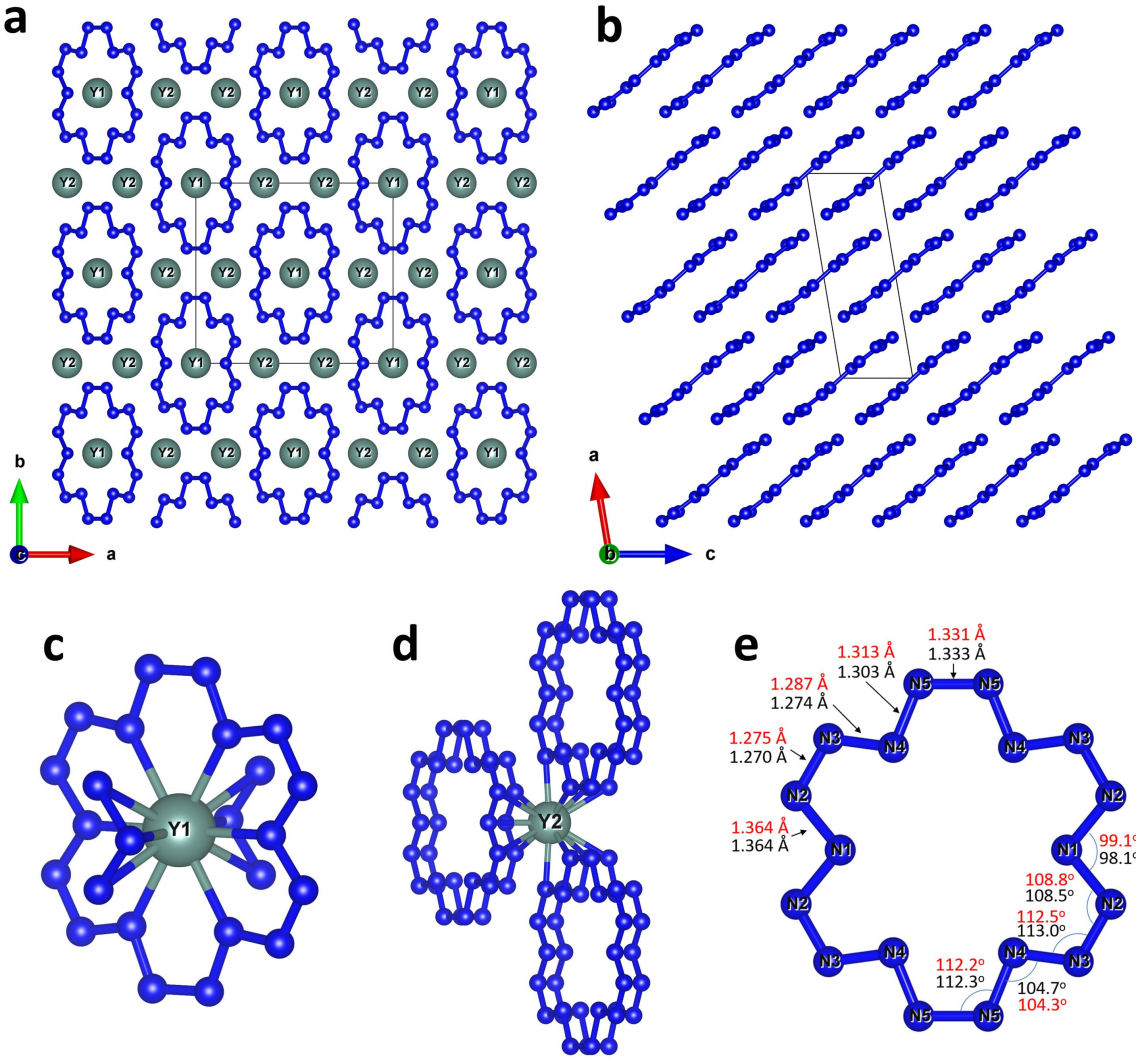
Crystal structure of YN_6_. All Y atoms are greenish, N atoms are blue; grey thin lines outline the unit cell. a) A view of the crystal structure along the *c*‐axis. b) A view of the crystal structure along the *b*‐axis; yttrium atoms are omitted. c) The coordination environment of the Y1 and d) Y2 atoms. e) A view of a N_18_ macrocycle; values of bond lengths and angles obtained from the experiment are shown in black, while those obtained from the DFT calculations are shown in red.

Although 2D polynitrogen layers composed of fused N_18_ rings were predicted for YN_8_ ^[37]^ and K_2_N_16_,^[44]^ the isolated anionic N_18_ rings observed in YN_6_ have never been reported from experiments or calculations. Hypothetically, a non‐charged N_18_ ring would be an aromatic planar ring with 18 electrons in the conjugated π‐system with the N−N bond order of 1.5. However, in the case of the YN_6_ compound, assuming +3 charge of yttrium atoms, the π‐system of each N_18_ ring should accommodate additional 9 electrons at the π* orbitals, which results in 27 electrons in the π‐system, losing aromaticity and thereby decreasing the average N−N bond order to 1.25 and consequently increasing of the N−N bond length. Moreover, the N_18_ rings in YN_6_ are non‐planar (the biggest torsion angle is 6.2(6)°), not satisfying the aromaticity condition. The N−N distances (*d*_N−N_) in the N_18_ macrocycle obtained from the SCXRD data are not equal and vary from 1.270(5) Å to 1.364(7) Å with the average *d*_N−N_=1.306(9) Å, in good agreement with the values obtained from the DFT‐relaxed structure (Figure 2e). The analysis of N−N bond lengths suggests that N1−N2 and N5−N5 are single bonds (single N−N bonds vary from 1.30 to 1.44 Å at ≈100 GPa[^24^, ^27^, ^29^, ^45^]), N2−N3 and N3−N4 have a bond order of 1.5 (the length of N−N bonds with the multiplicity of 1.5 is known to be in the range of 1.24 to 1.30 Å at ≈100 GPa^[25]^), while the multiplicity of the N4−N5 bond should be in between of 1 and 1.5. If one assumes the bond order of 1.125 for the N4−N5 bond, the average bond order in the [N_18_]^9−^ ring is of 1.25, which corresponds to 27 electrons in the π‐system and a total 9‐ charge. Non‐integer charges and bond orders were previously reported in metallic nitrides under high pressure.[^2^, ^7^, ^8^, ^26^]

Although 18‐membered cycles are not common, they are known for a number of organic compounds. As an example, one can mention 18‐crown‐6 ether, whose molecule is non‐planar as all ring‐forming atoms are sp^3^‐hybridized. A closer analog to the N_18_ rings we observed in YN_6_ would be an unsaturated cyclooctadecanonaene C_18_H_18_ ([18]annulene). The [18]annulene C_18_H_18_ is a fully planar aromatic compound, and until now the most nitrogen‐substituted known derivative is hexaaza[18]annulene C_12_N_6_H_12._ ^[46]^ The existence of [N_18_]^9−^ anionic macrocycle allows us to assume that high‐pressure could provide a route to the synthesis of N_18_ aromatic molecule–full nitrogen substituted [18]annulene derivative.

The Y_2_N_11_ compound, also synthesized at 100(1) GPa, crystallizes in the structure with the hexagonal space group *P*6_2_22 (#180), in which there are one Y and four N distinct atomic positions (see Table S2 and the CIF for the full crystallographic data^[40]^). Notably, the introduction of a racemic twinning law during the final structure refinement against SCXRD data leads to a slight decrease in the R_1_ factor, therefore, Y_2_N_11_ with the space group *P*6_2_22 coexists with its enantiomorph Y_2_N_11_ with the space group *P*6_4_22 (#181). The structure of Y_2_N_11_ (Figure 3) is built of Y atoms (CN=10) coordinated by discrete nitrogen atoms, N_2_ dumbbells, and polynitrogen chains (Figure 3c). Each discrete nitrogen atom is surrounded by four yttrium atoms forming regular tetrahedra NY_4_, which are connected through vertexes, forming a motif similar to the SiO_4_ tetrahedral motif in β‐quartz^[47]^ (Figure 3a, b). Like SiO_4_ groups in the structure of β‐quartz, the NY_4_ tetrahedra in Y_2_N_11_ form channels along the *c*‐axis (Figure 3a), which are occupied by nitrogen dumbbells with their centers on the 6_2_ axis (Figure 3a). The dumbbells themselves are also in a tetrahedral coordination environment of Y atoms (Figure 3d). Two polymeric nitrogen chains are running along the channels in the *c*‐direction (shown in blue in Figure 3a, b, e) forming a double helix arrangement (highlighted in blue and red in Figure 3f) around the 6_2_ screw‐axis. The crystal‐chemical formula of Y_2_N_11_ may be written as Y_2_N(N_4_)_2_(N_2_).

**Figure 3:**
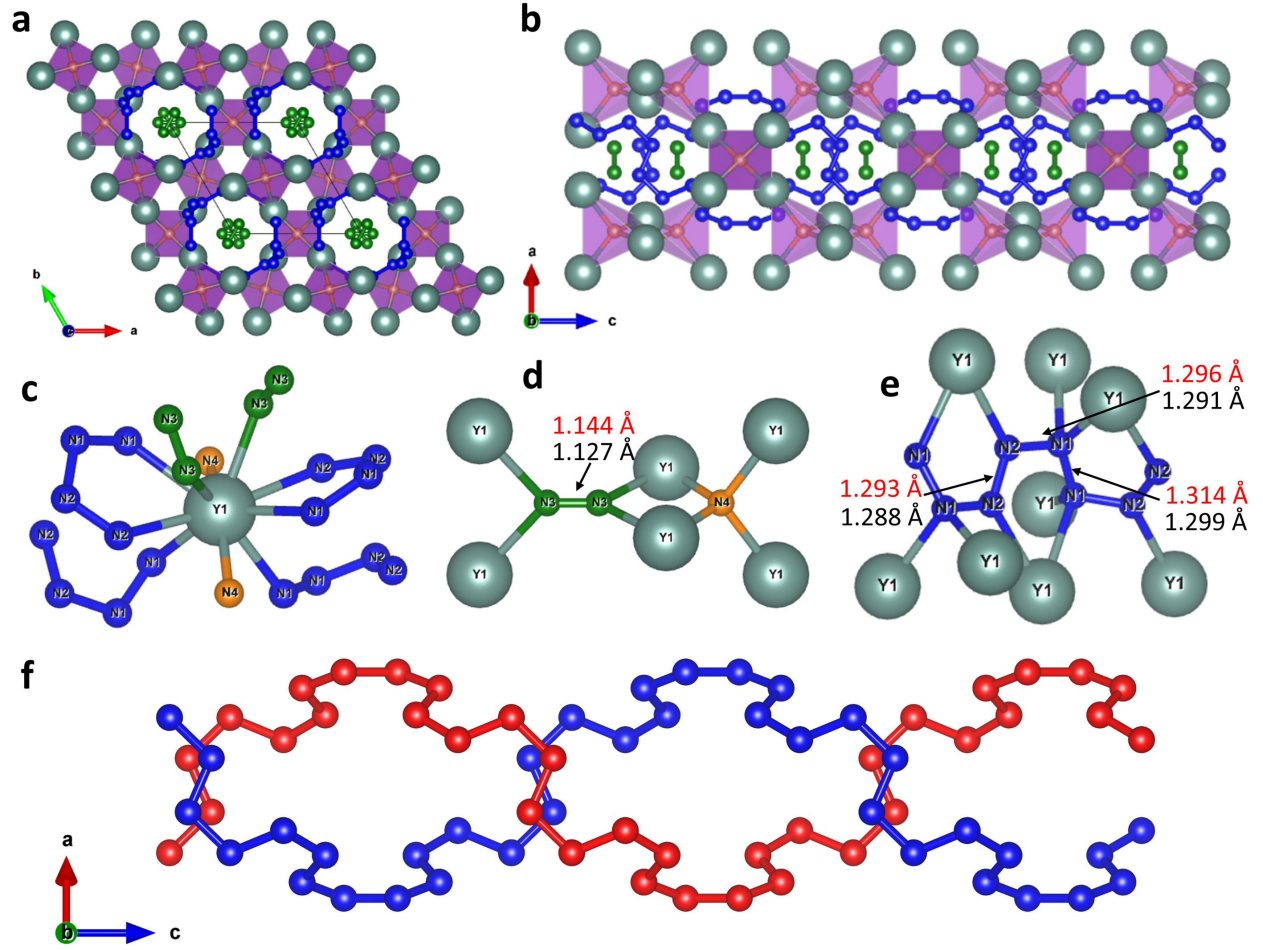
Crystal structure of Y_2_N_11_. All Y atoms are greenish, blue balls represent nitrogen atoms of the infinite chains, green balls—nitrogen atoms that form dumbbells, orange balls—discrete nitrogen atoms; grey thin lines outline the unit cell. a) A view of the crystal structure along the *c*‐axis. b) A view of the crystal structure along the *b*‐axis. c) The coordination environment of the Y atom. d) The coordination environment of discrete nitrogen atoms and nitrogen dumbbells. e) The coordination environment of polymeric nitrogen chain; values of bond lengths obtained from the experiment are shown in black, while those obtained from the DFT calculations are shown in red. f) Double helix built of two polynitrogen chains running along the *c*‐direction around the 6_2_ screw‐axis.

It appears that Y_2_N_11_ is isostructural to previously reported Hf_2_N_11_ (space group *P*6_4_22) synthesized by laser‐heating Hf in nitrogen at 105 GPa.^[26]^ The difference in the oxidation states of the cations Y^3+^ and Hf^4+^ in the isostructural compounds should lead to the difference in charge states of the corresponding nitrogen units. Indeed, taking into account that in the Hf_2_N_11_ structure at 105 GPa the N−N distance in N_2_ dimers is equal to 1.186 Å, and the average N−N distance in polynitrogen chains is 1.32 Å, the charge distribution was proposed as (Hf^4+^)_2_N^3−^(N_4_^2−^)_2_(N_2_^−^).^[26]^ In Y_2_N_11_ at 100(1) GPa, the N−N distance in N_2_ dimers is 1.13 Å 1.127(15), and the average N−N distance in polynitrogen chains is 1.29 Å, noticeably shorter than those in Hf_2_N_11_, indicating smaller charges on the nitrogen species. Since the N−N distance in N_2_ dimers is longer than that in the triple‐bonded non‐charged N_2_ molecule, but shorter than the bond length in N_2_^−^, one can suggest the N_2_^0.5−^ charge state for the dumbbells. Contrary to Hf_2_N_11_, the N−N distances in the polynitrogen chains in Y_2_N_11_ are almost equal and the length corresponds to a bond order between 1 and 1.5. Thus, one can assume the following charge distribution: (Y^3+^)_2_N^3−^(N_4_^1.25−^)_2_(N_2_^0.5−^). This example demonstrates that the π‐system of polynitrogen chains is flexible in terms of charge accommodation.

Inorganic double helixes are extremely rare[^48^, ^49^, ^50^] and the fact that such an arrangement of nitrogen atoms in the compounds was observed for the second time at about 100 GPa may indicate the existence of such a class of polynitrides under high pressure. Furthermore, the structure of Y_2_N_11_ can be considered as a host–guest, where yttrium and discrete nitrogen atoms form a β‐quartz‐type host framework (Figure 3), where inside and around the channels are guest nitrogen dimers and a polynitrogen double helix. Therefore, perhaps β‐quartz‐motifs could serve as a template for the synthesis of other inorganic double helixes.

In order to get a deeper insight into the chemistry and physical properties of the novel compounds, further DFT‐based calculations were performed (see Supporting Information for details). As mentioned above, variable‐cell structural relaxations for both compounds at the synthesis pressure closely reproduced structural parameters and bond lengths obtained from the experimental data. Phonon dispersion relations calculated in harmonic approximation show that both YN_6_ and Y_2_N_11_ phases are dynamically stable at 100 GPa (Figure 4a and Figure 4d). To estimate the thermodynamic stability of the novel phases, a static enthalpy convex‐hull for all known yttrium nitrides was calculated at 100 GPa. Within the used approximations, YN_6_ lies on the convex hull together with YN and Y_5_N_14_, while Y_2_N_11_ lays 189 meV per atom above the convex hull (Figure S2). Being smaller than k_B_T at synthesis temperature (3000 K, 258 meV), this suggests that the structure represents a local minimum in the potential energy landscape at synthesis conditions which is preserved as a meta‐stable state under rapid T‐quench to room temperature.

**Figure 4:**
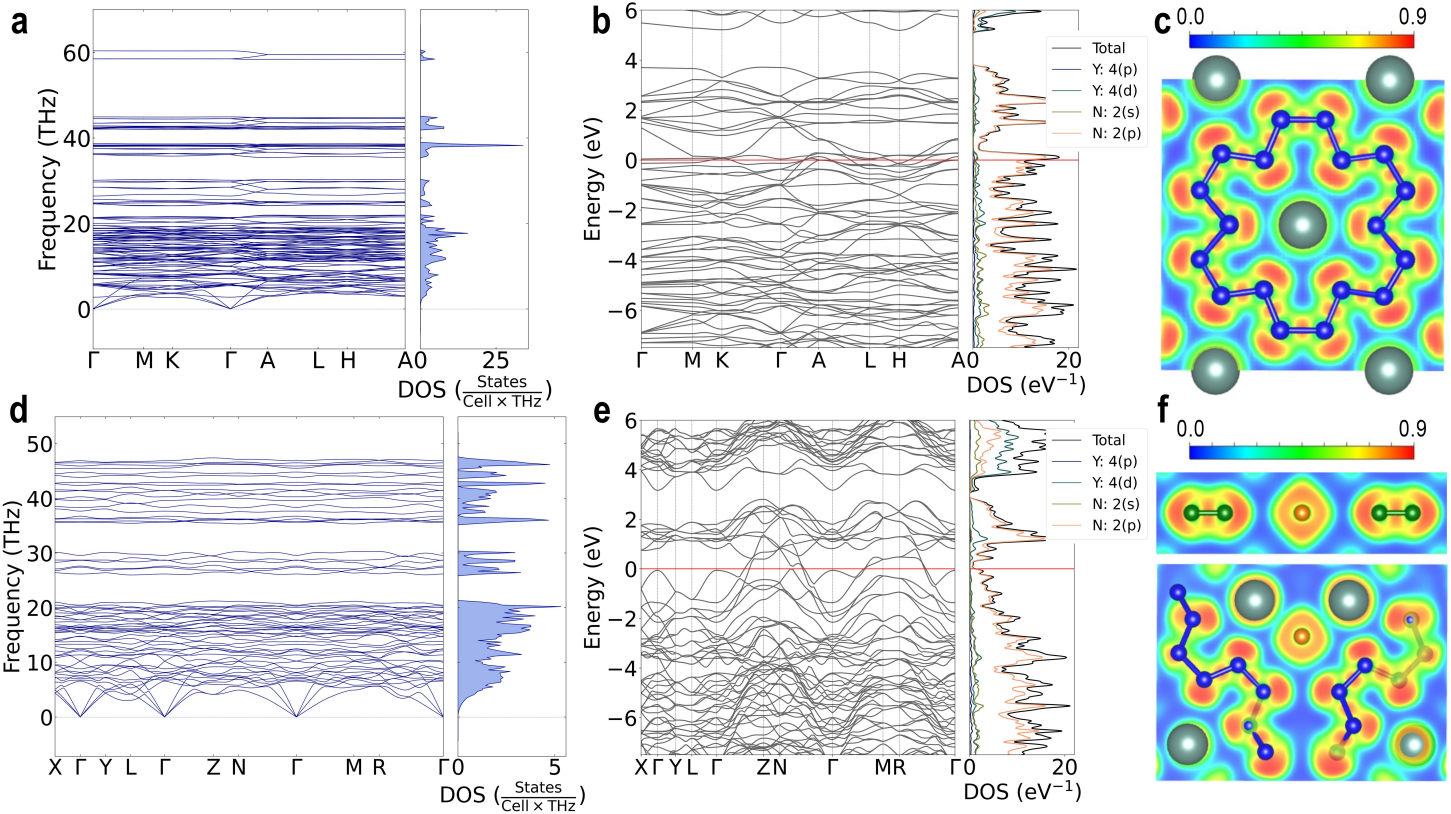
Calculated properties of YN_6_ and Y_2_N_11_ at 100 GPa. YN_6_: a) Phonon dispersions, b) electron density of states (red line indicates the Fermi level), c) electron localization function calculated in (4 0 ‐1) plane. Y_2_N_11_: d) The phonon dispersions, e) the electron density of states (red line indicates a Fermi level), f) the electron localization function calculated in (0 0 1) plane (upper figure) or in (3 ‐2 0) plane (bottom figure).

The agreement between theory and experiment at the synthesis pressure created the basis for the further analysis of chemistry and physical properties. To obtain an equation of state for YN_6_ and Y_2_N_11_, variable‐cell structure relaxations for both compounds were performed at target pressures between 0–150 GPa (using 10 GPa steps). Both structures were found to keep their respective symmetry in the relaxations within the full pressure range considered, however, the calculated phonon dispersion curves of YN_6_ and Y_2_N_11_ at atmospheric pressure show imaginary modes indicating dynamical instability at *T*=0 K (Figures S3 and S4). A 3^rd^ order Birch–Murnaghan equation of state was subsequently fitted to the obtained energy‐volume‐points (see Supporting Information). The obtained bulk moduli are lower than the bulk moduli of other experimentally known yttrium nitrides, YN and Y_5_N_14_, and the bulk modulus decreases with the decrease of yttrium content: *K*_0_(YN)=151.0 GPa^[51]^ > *K*_0_(Y_5_N_14_)=137.0 GPa^[8]^ > *K*_0_(Y_2_N_11_)=115.7 GPa > *K*_0_(YN_6_)=92.6 GPa. It is also worth noting that the bulk modulus of Y_2_N_11_ is significantly lower than the bulk modulus of the isostructural Hf_2_N_11_ compound (*K*_0_(Hf_2_N_11_)=193 GPa^[26]^) due to the higher compressibility of M^3+^−N bonds in Y_2_N_11_ compared to M^4+^−N bonds in Hf_2_N_11_.

The calculated electron localization functions for YN_6_ and Y_2_N_11_ at 100 GPa demonstrate a strong covalent bonding between nitrogen atoms in the N_18_ macrocycles, polynitrogen chains and nitrogen dimers (Figure 4c, f). At the same time, there is no obvious sign of covalent bonding between nitrogen and yttrium atoms and there is no sign of electron localization between these atoms, as expected for electrides.^[52]^ The N−N bond orders estimated from the crystal‐chemical analysis were corroborated by calculated crystal orbital bond index^[53]^ (Tables S4 and S5). The computed electron density of states shows that both phases are metals (Figure 4b and Figure 4e). Remarkably, the main electronic contribution at the Fermi level comes from the nitrogen *p*‐states, with an almost negligible contribution from yttrium states. The metallicity through the nitrogen π‐system explains the observation of non‐standard charges and bond multiplicities in the nitrogen units. Worth noting is also that the anion‐driven metallicity was found for the majority of high‐pressure di‐ and poly‐nitrides,[^2^, ^7^, ^8^, ^25^, ^26^, ^54^] which, therefore, seems to be a regular phenomenon for high‐pressure nitrides.

In conclusion, at 1 Mbar pressure, we have discovered two novel yttrium polynitrides YN_6_ and Y_2_N_11_, which are built from extremely unique nitrogen structural units: N_18_ macrocycles and double helix polynitrogen chains, respectively. The discovery of such compounds encourages further exploration of the remarkable inorganic chemistry of polynitrides. Moreover, the ability of nitrogen to form such structural units shows that perhaps humanity is on the verge of opening a new branch of chemistry—nitrogen organic chemistry under ultrahigh pressure.

## Conflict of interest

The authors declare no conflict of interest.

## Supporting information

As a service to our authors and readers, this journal provides supporting information supplied by the authors. Such materials are peer reviewed and may be re‐organized for online delivery, but are not copy‐edited or typeset. Technical support issues arising from supporting information (other than missing files) should be addressed to the authors.

Supporting Information

Supporting Information

Supporting Information

## Data Availability

The data that support the findings of this study are available in the Supporting Information of this article.
