# Supplementary material for: Anionic N18 Macrocycles and a Polynitrogen Double Helix in Novel Yttrium Polynitrides YN6 and Y2N11 at 100 GPa
Source: Angew Chem Int Ed Engl. 2022 Jul 13;61(34):e202207469. doi: 10.1002/anie.202207469 (PMC9546263; doi:10.1002/anie.202207469)
Supplement: Supplementary file 1 — Supporting Information [file ANIE-61-0-s003.pdf]

Angewandte Chemie
Eine Zeitschrift der Gesellschaft Deutscher Chemiker

Supporting Information

Anionic N_{18} Macrocycles and a Polynitrogen Double Helix in Novel Yttrium Polynitrides YN_6 and Y_2N_{11} at 100 GPa

A. Aslandukov, F. Trybel, A. Aslandukova, D. Laniel, T. Fedotenko, S. Khandarkhaeva, G. Aprilis, C. Giacobbe, E. Lawrence Bright, I. A. Abrikosov, L. Dubrovinsky, N. Dubrovinskaia*

Table of Contents

Experimental Procedures 2
Results 3
References 8

Experimental Procedures

Sample preparation. The BX90-type large X-ray aperture DAC[1] equipped with Boehler-Almax type diamonds[2] (culet diameter is 120 µm) was used in the experiment. The sample chamber was formed by pre-indenting of a rhenium gasket to ~20 µm thickness and drilling a hole of ~60 µm in diameter in the center of the indentation. Two pieces of yttrium (99.9%, Sigma Aldrich) were placed in a sample chamber, then molecular nitrogen was loaded using a high-pressure gas loading system (1300 bars).[3] The sample was compressed to ~100 GPa and laser-heated (λ = 1064 nm) up to 3000(200) K using the home-made double-sided laser-heating system at BGI,[4] as well as the double-sided laser heating system of ID18 beamline of the ESRF (Grenoble, France).[5] The temperature during laser-heating was determined by the blackbody radiation fit. The pressure in the cell was determined using the equation of state of Re[6] and additionally monitored by the Raman signal from the diamond anvils.[7]

X-ray diffraction. XRD measurements were performed at ID11 beamline of the ESRF (Grenoble, France) with the X-ray beam (λ = 0.2852 Å) focused down to 0.5 × 0.5 μm^2, and the XRD patterns were collected on a Eiger2X CdTe 4M hybrid photon counting pixel detector. For single-crystal XRD measurements, the sample was rotated around a vertical ω axis in a range of ±36°. The XRD images were collected with an angular step of Δω = 0.25° and an exposure time of 3s/frame. The $CrysAlis^{Pro}$ software package was used for the analysis of the single-crystal XRD data (peak hunting, indexing, data integration, frame scaling, and absorption correction). To calibrate the instrumental model in the $CrysAlis^{Pro}$ software, *i.e.* the sample-to-detector distance, detector's origin, offsets of the goniometer angles, and rotation of both the X-ray beam and detector around the instrument axis, we used a single crystal of orthoenstatite [$(Mg_{1.93}Fe_{0.06})(Si_{1.93},Al_{0.06})O_6$, *Pbca* space group, a = 8.8117(2) Å, b = 5.18320(10) Å, and c = 18.2391(3) Å]. The DAFi program[8] was used for the search of reflections' groups belonging to individual single-crystal domains. Using the OLEX2 software package,[9] the structures were solved with the ShelXT structure solution program[10] using intrinsic phasing and refined with the ShelXL[11] refinement package using least-squares minimization. Crystal structure visualization was made with the VESTA software.[12]

Density functional theory calculations. Kohn-Sham density function theory based structural relaxations and electronic structure calculations were performed with the QUANTUM ESPRESSO package[13–15] using the projector augmented wave approach.[16] We used the generalized gradient approximation by Perdew-Burke-Ernzerhof (PBE)[17] for exchange and correlation, with the corresponding potential files: for Y the 3d electrons and lower and for N the 1s electrons are treated as scalar-relativistic core states. Convergence tests with a threshold of 1 meV per atom in energy and 1 meV/Å per atom for forces led to a Monkhorst-Pack[18] k-point grid of 8x8x24 for YN_6, 12x12x12 for Y_2N_{11}, 8x8x16 for Y_5N_{14}, 8x8x8 for YN,16x16x16 for *cg*-N, and 8x8x3 for hR24-Y. For all calculations a cutoff for the wave-function expansion of 80 Ry and a gaussian smearing of 0.005 Ry was used. Phonon calculations were performed with PHONOPY[19] in 2x2x2 supercells with respectively adjusted k-points. The crystal orbital bond index (COBI)[20] was calculated using LOBSTER v4.1.0 software[21].

We performed variable cell relaxations (lattice parameters and atomic positions) on all experimental structures to optimize the atomic coordinates and the cell vectors until the total forces were smaller than 10^{-4} eV/Å per atom and the deviation from the experimental pressure was below 0.1 GPa. Furthermore, we calculated equation of states (EOS) by performing variable cell relaxations to respective target pressures until forces are < 10^{-3} eV/Å and pressure is matched within 0.1 GPa. A third order Birch Murnagham EOS was fitted to the calculated energy versus volume points. We obtained

YN_6:	K_0 = 92.6 GPa,	K' = 5.13,	V_0 = 423.59 $Å^3$
Y_2N_{11}:	K_0 = 115.7 GPa,	K' = 4.38,	V_0 = 401.48 $Å^3$

Results

Table S1. Structure refinement details of YN_6 at 100(1) GPa. The full crystallographic dataset was deposited to the CCDC under the deposition number 2173746.

Chemical formula	YN_6
Temperature (K)	293
Pressure (GPa)	100(1)
**Crystal data**	
Mr	172.97
ρ (g/cm^3)	6.180
Crystal system, space group	monoclinic, *C*2/*c*
a (Å)	9.667(3)
b (Å)	8.7071(15)
c (Å)	3.3592(9)
β (°)	99.52(3)
V (Å^3)	278.86(12)
Z	6
Radiation type	X-ray, λ = 0.2852 Å
μ (mm^{-1})	2.823
**Data collection**	
No. of measured, independent and observed [I > 2σ(I)] reflections	918/648/526
R_{int}	1.17%
(sin θ/λ)$_{max}$ (Å^{-1})	1.205
**Refinement**	
R[F^2 > 3σ(F^2)], wR(F^2), GOF	0.0470, 0.1270, 1.023
data/parameters ratio	648/53
$\Delta\rho_{max}$, $\Delta\rho_{min}$ (e Å^{-3})	2.83, -1.96

Atomic positions

Atom	Wyckoff site	Fractional atomic coordinates		
		x	y	z
Y1	2*a*	0	0	0
Y2	4*i*	0.34719(6)	0	0.28733(18)
N1	4*i*	0.1516(6)	0	0.5598(16)
N2	8*j*	0.3011(4)	0.3817(3)	0.1879(12)
N3	8*j*	0.3391(4)	0.2562(4)	0.3655(12)
N4	8*j*	0.0804(5)	0.2245(3)	0.2976(12)
N5	8*j*	0.0469(5)	0.3630(3)	0.1695(12)

Anisotropic displacement parameters

Atom	U_{11} (Å^2)	U_{22} (Å^2)	U_{33} (Å^2)	U_{12} (Å^2)	U_{13} (Å^2)	U_{23} (Å^2)	U_{eq} (Å^2)
Y1	0.0114(4)	0.0047(2)	0.0117(3)	0	-0.0038(3)	0	0.00990(19)
Y2	0.0111(3)	0.0047(2)	0.0119(2)	0	-0.0039(2)	0	0.00991(16)
N1	0.012(3)	0.0069(16)	0.011(2)	0	-0.003(2)	0	0.0105(12)
N2	0.009(2)	0.0063(11)	0.0112(13)	-0.0002(7) -	-0.0039(13)	-0.0003(7)	0.0096(8)
N3	0.010(2)	0.0070(13)	0.0148(17)	0.0004(7)	-0.0028(16)	0.0000(7)	0.0112(9)
N4	0.0108(19)	0.0062(11)	0.0132(15)	0.0020(7)	-0.0046(14)	0.0002(7)	0.0108(8)
N5	0.012(2)	0.0068(12)	0.0129(15)	-0.0012(8) -	-0.0032(14)	0.0005(7)	0.0112(8)

Table S2. Structure refinement details of Y_2N_{11} at 100(1) GPa. The full crystallographic dataset was deposited to the CCDC under the deposition number 2173750.

Chemical formula	Y_2N_{11}
Temperature (K)	293
Pressure (GPa)	100(1)
**Crystal data**	
Mr	331.93
ρ (g/cm^3)	6.104
Crystal system, space group	hexagonal, $P6_222$
a (Å)	6.478(4)
c (Å)	7.454(6)
V (Å^3)	270.9(4)
Z	3
Radiation type	X-ray, λ = 0.2852 Å
μ (mm^{-1})	2.900
**Data collection**	
No. of measured, independent and observed [I > 2σ(I)] reflections	1111/744/551
R_{int}	4.94%
$(\sin θ/λ)_{max}$ (Å^{-1})	1.222
**Refinement**	
R[F^2 > 3σ(F^2)], wR(F^2), GOF	0.0517, 0.1278, 1.004
data/parameters ratio	685/32
$Δρ_{max}$, $Δρ_{min}$ (e Å^{-3})	1.73, -1.85

Atomic positions

Atom	Wyckoff site	Fractional atomic coordinates		
		x	y	z
Y1	6*j*	0.20989(7)	0.41978(14)	0.5
N1	12*k*	0.2661(9)	0.4451(9)	0.0785(8)
N2	12*k*	0.1052(9)	0.3831(10)	0.2012(7)
N3	6*g*	0.0870(11)	0	0
N4	3*c*	0.5	0	0

Anisotropic displacement parameters

Atom	U_{11} (Å^2)	U_{22} (Å^2)	U_{33} (Å^2)	U_{12} (Å^2)	U_{13} (Å^2)	U_{23} (Å^2)	U_{eq} (Å^2)
Y1	0.00700(18)	0.0076(2)	0.0107(2)	0.00379(12)	-0.00008(18)	0	0.00836(14)
N1	0.0077(15)	0.0067(16)	0.0097(17)	0.0032(14)	-0.0004(12)	-0.0001(13)	0.0082(7)
N2	0.0066(16)	0.011(2)	0.0086(19)	0.0042(14)	0.0023(12)	-0.0003(13)	0.0090(8)
N3	0.0088(16)	0.007(2)	0.015(3)	0.0033(10)	0.0016(12)	0.003(2)	0.0104(10)
N4	0.0027(16)	0.008(3)	0.007(3)	0.0040(15)	0	0	0.0054(11)

Table S3. Experimentally determined crystallographic data for the YN_6 and Y_2N_{11} phases at 100(1) GPa compared with the corresponding DFT-relaxed structures. Note that pressure was fixed in theoretical simulations, while the volume of the unit cells, lattice parameters and equilibrium state parameters were calculated.

	**YN_6**		**Y_2N_{11}**	
	**Exp.**	**Calc.**	**Exp.**	**Calc.**
**Space group**	*C*2/*c*	*C*2/*c*	*P*6$_2$22	*P*6$_2$22
**Volume**	278.86(12) Å^3	286.19 Å^3	270.9(4) Å^3	275.7 Å^3
**Lattice parameters**	a = 9.667(3) Å<br>b = 8.7071(15) Å<br>c = 3.3592(9) Å<br>α = 90°<br>β = 99.52(3)°<br>γ = 90°	a = 9.7902 Å<br>b = 8.7467 Å<br>c = 3.3938 Å<br>α = 90°<br>β = 100.01°<br>γ = 90°	a = 6.478(4) Å<br>b = 6.478(4) Å<br>c = 7.454(6) Å<br>α = 90°<br>β = 90°<br>γ = 120°	a = 6.4863 Å<br>b = 6.4863 Å<br>c = 7.5667 Å<br>α = 90°<br>β = 90°<br>γ = 120°
**Atomic positions**	Y1 x 0<br>y 0<br>z 0	Y1 x 0.00000<br>y 0.00000<br>z 0.00000	Y1 x 0.20989(7)<br>y 0.41978(14)<br>z 0.5	Y1 x 0.20842<br>y 0.41667<br>z 0.50000
	Y2 x 0.34719(6)<br>y 0<br>z 0.28733(18)	Y2 x 0.34799<br>y 0.00000<br>z 0.28283	N1 x 0.2661(9)<br>y 0.4451(9)<br>z 0.0785(8)	N1 x 0.26628<br>y 0.44517<br>z 0.07838
	N1 x 0.1516(6)<br>y 0<br>z 0.5598(16)	N1 x 0.15206<br>y 0.00000<br>z 0.56359	N2 x 0.1052(9)<br>y 0.3831(10)<br>z 0.2012(7)	N2 x 0.10570<br>y 0.38217<br>z 0.20044
	N2 x 0.3011(4)<br>y 0.3817(3)<br>z 0.1879(12)	N2 x 0.30237<br>y 0.38133<br>z 0.18711	N3 x 0.0870(11)<br>y 0<br>z 0	N3 x 0.08823<br>y 0.00000<br>z 0.00000
	N3 x 0.3391(4)<br>y 0.2562(4)<br>z 0.3655(12)	N3 x 0.33794<br>y 0.25598<br>z 0.36799	N4 x 0.5<br>y 0<br>z 0	N4 x 0.50000<br>y 0.00000<br>z 0.00000
	N4 x 0.0804(5)<br>y 0.2245(3)<br>z 0.2976(12)	N4 x 0.07877<br>y 0.22361<br>z 0.29871		
	N5 x 0.0469(5)<br>y 0.3630(3)<br>z 0.1695(12)	N5 x 0.04678<br>y 0.36250<br>z 0.16765		

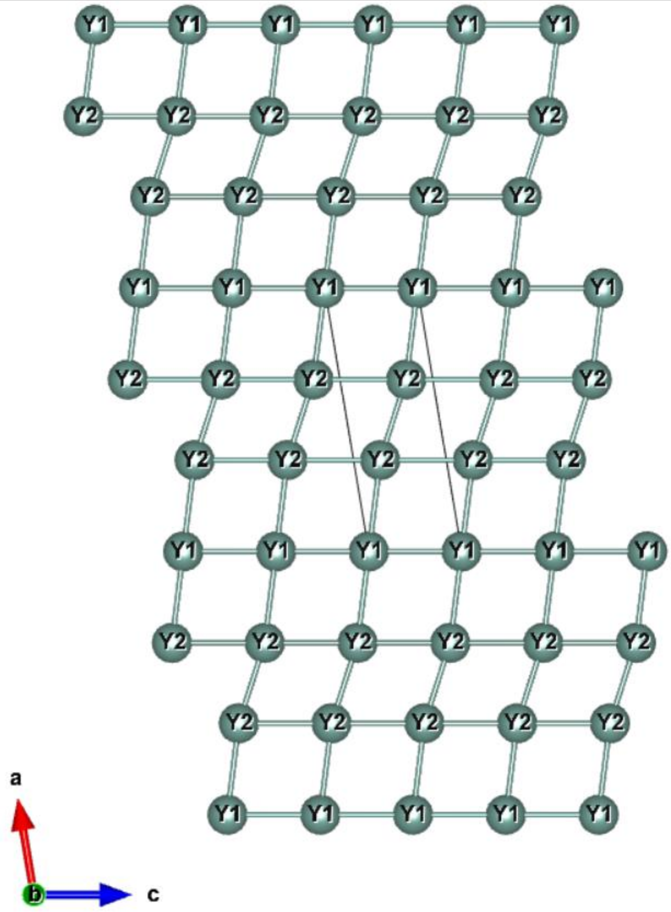

Figure S1. A layer of Y atoms in the *ac* plane in the YN_6 structure.

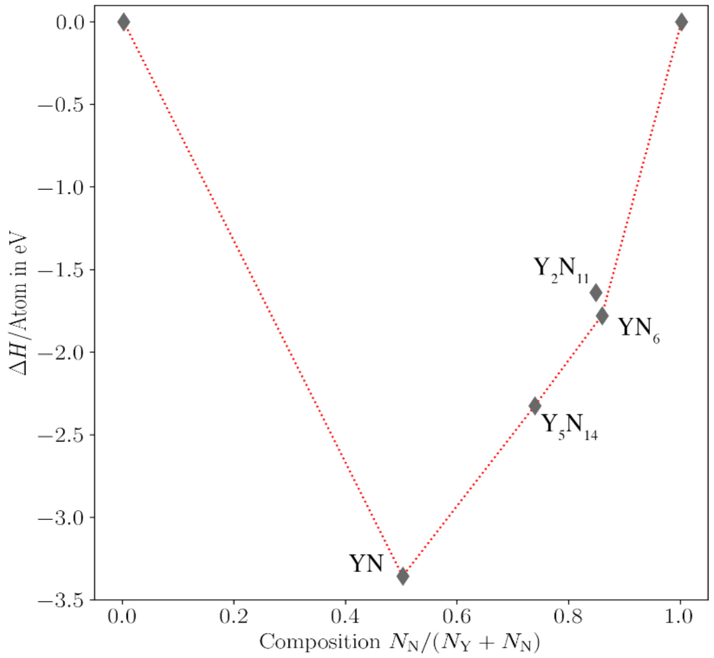

Figure S2. The calculated convex hull in the Y-N binary system for known yttrium nitrides at 100 GPa. Static enthalpies were calculated for Y (*hR*24 structure[22]), nitrogen (*cg*-N structure[23]), YN (rocksalt structure), Y_5N_{14}[24] and two compounds, YN_6 and Y_2N_{11}, from the current study. The phases YN, Y_5N_{14} and YN_6 lie on the convex hull.

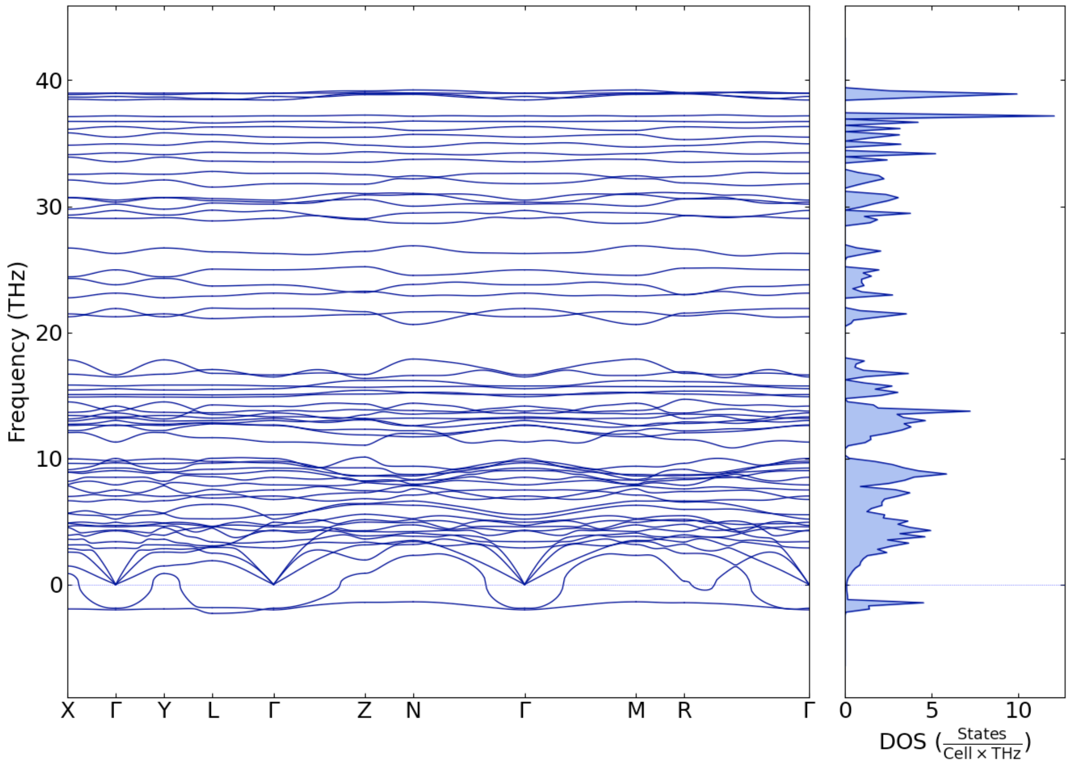

Figure S3. Phonon dispersions of YN_6 at 1 bar.

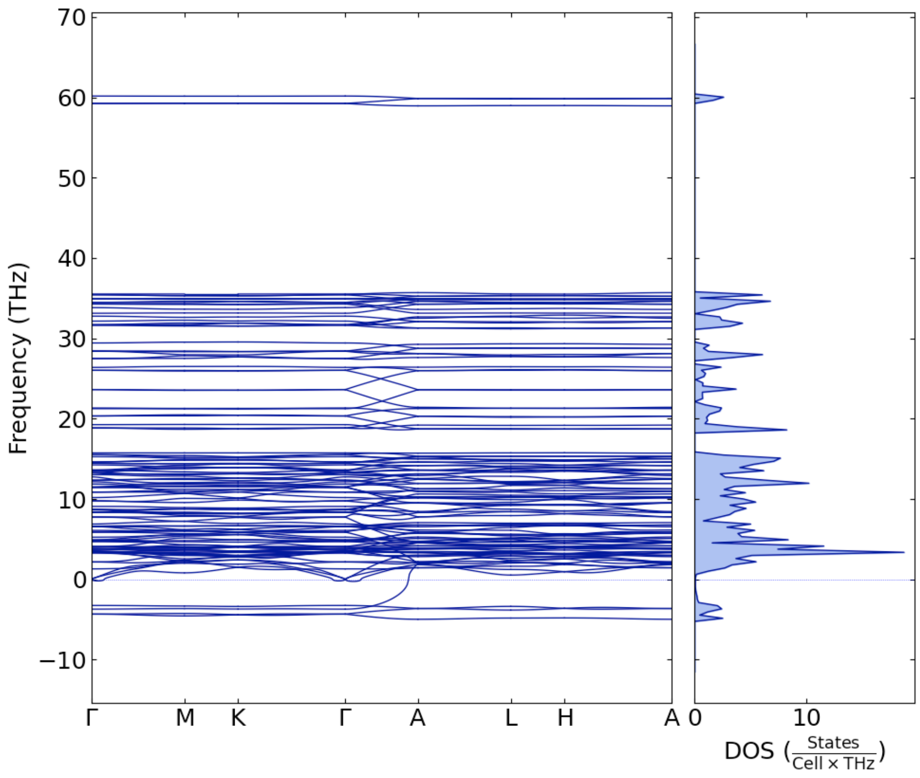

Figure S4. Phonon dispersions of Y_2N_{11} at 1 bar.

Table S4. Crystal orbital bond index of nitrogen-nitrogen bonds in N_{18} macrocycle in YN_6

Bond	Bond length	Bond index COBI	Bond order estimated from crystal-chemical analysis
N1-N2	1.364	0.996	1
N2-N3	1.275	1.232	1.5
N3-N4	1.287	1.256	1.5
N4-N5	1.313	1.122	1.125
N5-N5	1.331	1.043	1

Table S5. Crystal orbital bond index of nitrogen-nitrogen bonds in polynitrogen chains and nitrogen dimers in Y_2N_{11}

Bond	Bond length	Bond index COBI	Bond order estimated from crystal-chemical analysis
N1-N1 (chains)	1.315	1.092	between 1 and 1.5
N1-N2 (chains)	1.296	1.184	between 1 and 1.5
N2-N2 (chains)	1.293	1.242	between 1 and 1.5
N3-N3 (dimers)	1.145	2.051	2.75

References

[1] I. Kantor, V. Prakapenka, A. Kantor, P. Dera, A. Kurnosov, S. Sinogeikin, N. Dubrovinskaia, L. Dubrovinsky, *Rev. Sci. Instrum.* **2012**, *83*, 125102.
[2] R. Boehler, *Rev. Sci. Instrum.* **2006**, *77*, 2004–2007.
[3] A. Kurnosov, I. Kantor, T. Boffa-Ballaran, S. Lindhardt, L. Dubrovinsky, A. Kuznetsov, B. H. Zehnder, *Rev. Sci. Instrum.* **2008**, *79*, 045110.
[4] T. Fedotenko, L. Dubrovinsky, G. Aprilis, E. Koemets, A. Snigirev, I. Snigireva, A. Barannikov, P. Ershov, F. Cova, M. Hanfland, N. Dubrovinskaia, *Rev. Sci. Instrum.* **2019**, *90*, 104501.
[5] I. Kupenko, L. Dubrovinsky, N. Dubrovinskaia, C. McCammon, K. Glazyrin, E. Bykova, T. B. Ballaran, R. Sinmyo, A. I. Chumakov, V. Potapkin, A. Kantor, R. Rüffer, M. Hanfland, W. Crichton, M. Merlini, *Rev. Sci. Instrum.* **2012**, *83*, 124501.
[6] S. Anzellini, A. Dewaele, F. Occelli, P. Loubeyre, M. Mezouar, *J. Appl. Phys.* **2014**, *115*, 043511.
[7] Y. Akahama, H. Kawamura, *J. Appl. Phys.* **2006**, *100*, 043516.
[8] A. Aslandukov, M. Aslandukov, N. Dubrovinskaia, L. Dubrovinsky, *J. Appl. Crystallogr.* **2022**, under review.
[9] O. V. Dolomanov, L. J. Bourhis, R. J. Gildea, J. A. K. Howard, H. Puschmann, *J. Appl. Crystallogr.* **2009**, *42*, 339–341.
[10] V. Petrícek, M. Dušek, L. Palatinus, *Zeitschrift fur Krist.* **2014**, *229*, 345–352.
[11] G. M. Sheldrick, *Acta Crystallogr. Sect. C Struct. Chem.* **2015**, *71*, 3–8.
[12] K. Momma, F. Izumi, *J. Appl. Crystallogr.* **2011**, *44*, 1272–1276.
[13] P. Giannozzi, S. Baroni, N. Bonini, M. Calandra, R. Car, C. Cavazzoni, D. Ceresoli, G. L. Chiarotti, M. Cococcioni, I. Dabo, A. Dal Corso, S. De Gironcoli, S. Fabris, G. Fratesi, R. Gebauer, U. Gerstmann, C. Gougoussis, A. Kokalj, M. Lazzeri, L. Martin-Samos, N. Marzari, F. Mauri, R. Mazzarello, S. Paolini, A. Pasquarello, L. Paulatto, C. Sbraccia, S. Scandolo, G. Sclauzero, A. P. Seitsonen, A. Smogunov, P. Umari, R. M. Wentzcovitch, *J. Phys. Condens. Matter* **2009**, *21*, 395502.
[14] P. Giannozzi, O. Andreussi, T. Brumme, O. Bunau, M. B. Nardelli, M. Calandra, R. Car, C. Cavazzoni, D. Ceresoli, M. Cococcioni, others, *J. Phys. Condens. Matter* **2017**, *29*, 465901.
[15] P. Giannozzi, O. Baseggio, P. Bonfà, D. Brunato, R. Car, I. Carnimeo, C. Cavazzoni, S. De Gironcoli, P. Delugas, F. Ferrari Ruffino, A. Ferretti, N. Marzari, I. Timrov, A. Urru, S. Baroni, *J. Chem. Phys.* **2020**, *152*, 154105.
[16] P. E. Blöchl, *Phys. Rev. B* **1994**, *50*, 17953–17979.
[17] J. P. Perdew, K. Burke, M. Ernzerhof, *Phys. Rev. Lett.* **1996**, *77*, 3865–3868.
[18] H. J. Monkhorst, J. D. Pack, *Phys. Rev. B* **1976**, *13*, 5188–5192.
[19] A. Togo, I. Tanaka, *Scr. Mater.* **2015**, *108*, 1–5.
[20] P. C. Müller, C. Ertural, J. Hempelmann, R. Dronskowski, *J. Phys. Chem. C* **2021**, *125*, 7959–7970.
[21] R. Nelson, C. Ertural, J. George, V. L. Deringer, G. Hautier, R. Dronskowski, *J. Comput. Chem.* **2020**, *41*, 1931–1940.
[22] E. J. Pace, S. E. Finnegan, C. V. Storm, M. Stevenson, M. I. McMahon, S. G. MacLeod, E. Plekhanov, N. Bonini, C. Weber, *Phys. Rev. B* **2020**, *102*, 094104.
[23] M. I. Eremets, A. G. Gavriliuk, I. A. Trojan, D. A. Dzivenko, R. Boehler, *Nat. Mater.* **2004**, *3*, 558–563.
[24] A. Aslandukov, A. Aslandukova, D. Laniel, I. Koemets, T. Fedotenko, L. Yuan, G. Steinle-Neumann, K. Glazyrin, M. Hanfland, L. Dubrovinsky, N. Dubrovinskaia, *J. Phys. Chem. C* **2021**, *125*, 18077−18084.
